# Globally invasive populations of the clonal raider ant are derived from Bangladesh

**DOI:** 10.1098/rsbl.2020.0105

**Published:** 2020-06-17

**Authors:** Waring Trible, Sean K. McKenzie, Daniel J. C. Kronauer

**Affiliations:** 1 Laboratory of Social Evolution and Behavior, The Rockefeller University, 1230 York Avenue, New York, NY, 10065, USA; 2 Center for Systems Biology, Harvard University, 52 Oxford Street, NW 369.20, Cambridge, MA 02138, USA; 3 Department of Ecology and Evolution, University of Lausanne, Lausanne CH-1015, Switzerland

**Keywords:** clonality, Formicidae, invasion history, invasive species, *Ooceraea biroi*, thelytoky

## Abstract

Identifying the native range of invasive species is useful to understand their evolution and natural history, as well as to develop new methods to control potentially harmful introduced organisms. The clonal raider ant, *Ooceraea biroi*, is an introduced species and an increasingly important social insect model organism, but its native range remains unknown. Here, we report a new series of *O. biroi* collections from Bangladesh, Singapore, Vietnam and China. We use a molecular phylogeny constructed with five gene fragments from 27 samples to determine that invasive lineages of *O. biroi* originated in Bangladesh. These lineages may have spread from Bangladesh via the historically significant Bay of Bengal shipping ports. *Ooceraea biroi* shares multiple features of its biology with other introduced ants, including parthenogenesis, retention of heterozygosity and presence of multiple egg-layers in the colony. Using laboratory rearing and microsatellite markers, we show that colonies collected from disturbed habitat in Bangladesh have these traits in common with colonies from the invasive range. Ancestral populations with sexual reproduction in primary habitats either remain to be discovered or have gone extinct. Our findings advance our understanding of the global spread of the clonal raider ant and highlight a suite of general traits that make certain ants prone to becoming invasive.

## 1. Introduction

A number of tramp ant species have been spread by human commerce throughout the world. Studies of their native populations allow researchers to better understand the circumstances under which these species have evolved, identify general characteristics that predispose them to become invasive and uncover natural biological control agents that could limit their spread. Unfortunately, the exact native range and likely route of invasion remain unknown for the great majority of invasive species [1].

In recent years, genetic data have identified the source populations and likely invasion routes for a handful of invasive ants [2–5]. In the absence of strong confounding factors, the native range of a species is expected to contain more genetic diversity than the invasive range [6]. Furthermore, invasive genotypes will be phylogenetically nested within the diversity of native genotypes and will be more closely related to native genotypes from their source population than from geographically more distant native populations. For most invasive ants, however, these genetic signatures have not been reported, and precise source populations therefore remain unknown (e.g. [7–9]). Challenges arise when the global distribution of a species is poorly known, when species are difficult to collect and when putatively native populations are difficult to pinpoint or reside in inaccessible regions of the planet.

The clonal raider ant, *Ooceraea biroi*, is queenless, and colonies are composed of a few dozen to a few hundred unmated workers, all of which can reproduce via thelytokous parthenogenesis [10,11]. *Ooceraea biroi* reproduces via automixis with central fusion, where the two central meiotic products fuse after meiosis II, which, in the absence of recombination, restores the maternal genotype [11]. Parthenogenesis and the presence of several reproductively active females in a colony are overrepresented among introduced ant species and are believed to facilitate the establishment of small founding populations in new habitats [12–14]. Its unusual biology might therefore explain why *O. biroi* is currently the only known invasive species in the ant subfamily Dorylinae.

The clonal raider ant is known primarily from tropical and subtropical islands worldwide, where it was presumably introduced via human activity [10,15]. Additional localities reported since earlier reviews [10,15] include Pakistan [16], Sri Lanka [17], Macau [18] and Cuba [19].

Based on phylogenetic evidence and microsatellite markers, all assayed invasive colonies belong to one of four clonal lineages, termed Lines A, B, C and D, or to lineages that arose from rare mating events between invasive lines [10]. Four presumably native samples of *Ooceraea* from India, China and Vietnam have been determined to correspond either to genetically divergent lineages of *O. biroi* or to closely related species (genotypes E, F, G and H) [10,20,21]. Of these, a colony from Uttarakhand, India was the closest relative of invasive lines (genotype E; [10]). However, the native source population of Lines A, B, C and D remains unknown.

Subterranean invasive ants are mainly spread via soil, such as the ballast of ships [5]. We hypothesized that Bangladesh could be the source of invasive *O. biroi* populations, as this country neighbours eastern India and is host to the Bay of Bengal, a major Asian shipping port.

## 2. Methods

For additional details, see electronic supplementary material, Methods. Our final dataset consisted of 16 independent collections of *O. biroi* from Bangladesh, one colony from Shenzhen, China, one colony from Singapore and one colony from Ba Vì, Vietnam, in conjunction with previously published sequences. Phylogenetic analysis was conducted using five gene fragments (*cytochrome oxidase I* (*COI*), *cytochrome oxidase II* (*COII*), *wingless* (*wg*), *elongation factor 1α* (*EF1α*) and *long wavelength rhodopsin* (*LR*)) from a total of 27 independent samples (electronic supplementary material table S1).

Five conserved microsatellite loci from nine exemplar colonies (5–7 ants per colony) were used for population genetic analysis. Two criteria were employed to infer clonality in colonies collected from Bangladesh: (i) colonies could be maintained in the laboratory without mating (see details in §3) and (ii) genotypes across five microsatellite loci were consistent with clonal reproduction.

## 3. Results

From the 16 colonies we collected in Bangladesh, we recovered seven unique mitochondrial haplotypes across *COI* and *COII* sequences. Two of these were identical to those of known invasive lines, Lines C and D, and the remaining five had not been previously reported (electronic supplementary material, table S1). All microsatellite data were consistent with clonal reproduction (electronic supplementary material, table S2) [10]. As further support for asexual reproduction, we were able to maintain two of the new lines, I and L, in the laboratory for over five years without any evidence of mating. The maximum lifespan of *O. biroi* is *ca.* 1.5 years, implying that all of the individuals collected initially in the field had long died. Colonies that were not maintained in the laboratory were either collected with low numbers of individuals and/or died in captivity (for colony sizes in the field, see electronic supplementary material, table S1). Finally, we visually inspected all ants from each colony immediately upon collection and never observed any morphological queens. Queen production was neither observed in the laboratory.

**Figure 1.**
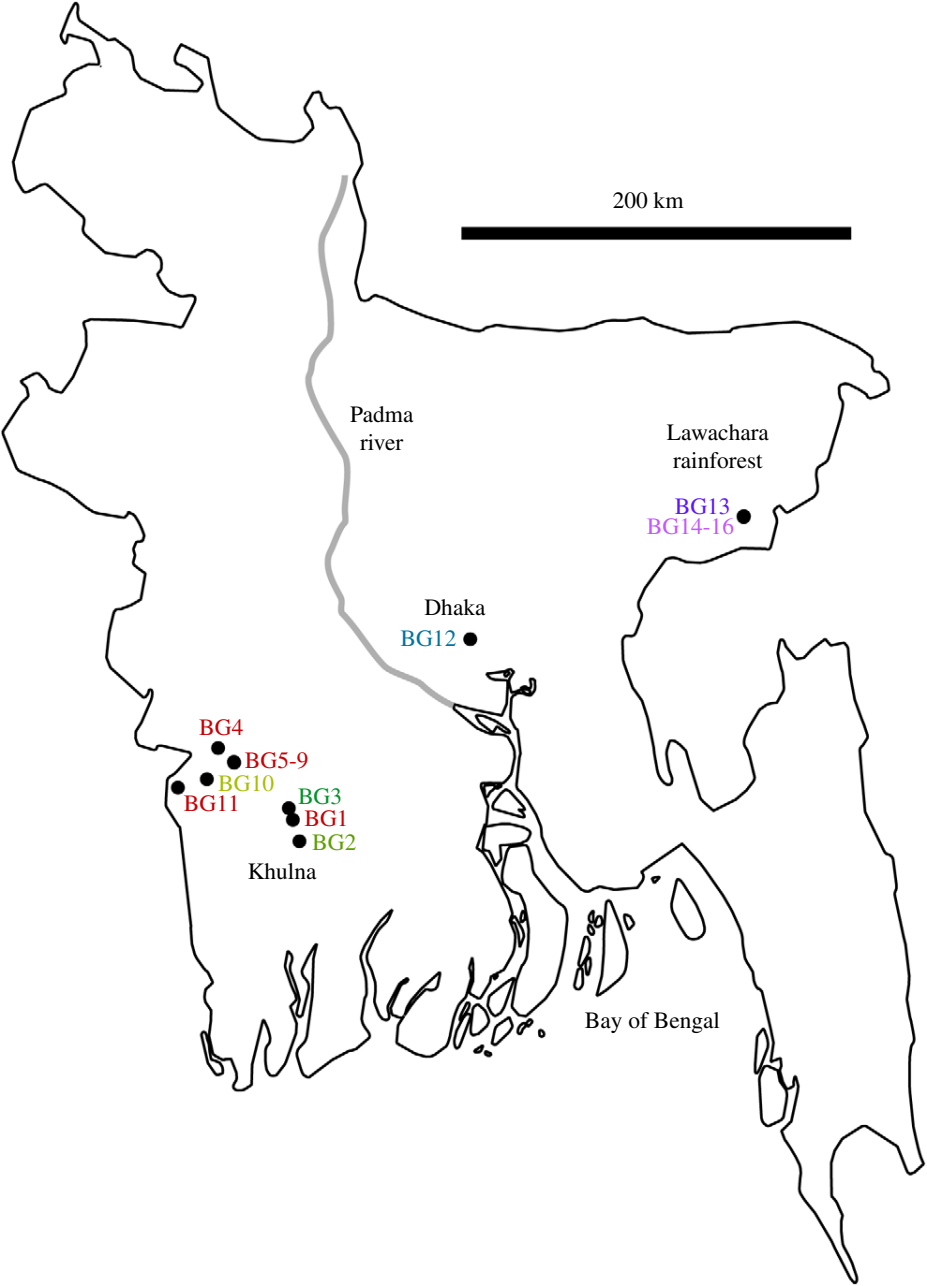
Collection localities for colonies from Bangladesh. Geographic coordinates and location names are given in electronic supplementary material, table S1.

Based on the totality of evidence, we conclude that the seven mitochondrial haplotypes we collected in Bangladesh correspond to at least seven unique clonal lines. Two of these belong to known invasive lines, Lines C and D, and we designate the other five as new lines, Lines I, J, K, L and M (electronic supplementary material, tables S1 and S2). This sample population encompasses the 600 km width of the country, and new lines were found in the vicinity of Khulna, Dhaka and the Lawachara Rainforest (figure 1).

**Figure 2.**
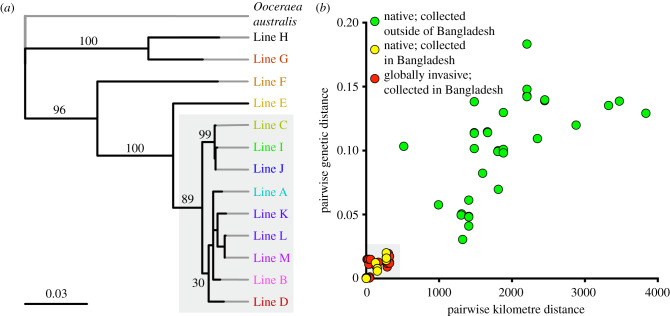
Phylogenetic analysis. (*a*) Phylogeny of *O. biroi* from Bangladesh and the globally invasive range. Numbers indicate bootstrap support; scale bar indicates proportional divergence at informative loci. Globally invasive lines (A–D) are nested within the diversity of Bangladesh lines (I–M; globally invasive lines C and D were also found in Bangladesh) (grey box). Outgroup in grey: *O. australis* (senior synonym of *Cerapachys edentata* [22]). (*b*) Relationship between geographic and genetic distances of *Ooceraea* collections from the Asian continent. Genetic distances are derived from branch lengths in figure 2*a*. Green points (native; collected outside of Bangladesh) are pairwise distances between every native Ooceraea collection from India, Vietnam and China with every other such collection, and with every Bangladesh line. Yellow points (native; collected in Bangladesh) are distances between all pairs of Bangladesh lines (I–M). Red points (globally invasive; collected in Bangladesh) represent distances between our new Bangladesh collections of globally invasive lines (C and D) and our new Bangladesh lines (I–M).

To test whether Bangladesh is the source of invasive lines, we performed a molecular phylogenetic analysis (figure 2*a*). The five new lines from Bangladesh are more closely related to the four invasive lines than the previously described lines from continental Asia. In fact, the invasive lines are phylogenetically nested within the genetic diversity of Bangladesh lines.

The distance matrix of this phylogeny reveals a linear association of genetic and geographic distances in native samples from the Asian continent (figure 2*b*, green). The globally invasive lines we collected in Bangladesh did not deviate from this relationship and were geographically and genetically close to our newly described Bangladesh lines (figure 2*b*; red and yellow, respectively). As an example, Lines I, J and C were found within a 50 km radius, are closely related and form a clade, whereas Line E, found 1300 km away in Uttarakhand, is genetically more distant and the sister to the clade of Bangladesh and invasive lines. This concordance of geographic and genetic distances indicates that the invasive lines are almost certainly derived from within Bangladesh.

Finally, we genotyped three additional new collections from Singapore, Shenzhen (China) and Ba Vì (Vietnam). These colonies belong to Lines B, C and D, respectively, confirming that invasive *O. biroi* lines have become established in Singapore and mainland Asia [15].

## 4. Discussion

Our results provide a number of new insights into the biology and invasion history of the clonal raider ant. With seven unique mitochondrial haplotypes among 16 sampled colonies, Bangladesh contains by far the most genetically diverse *O. biroi* population known to date. For example, [10] observed two mitochondrial haplotypes in 22 colonies from Okinawa, Japan, and just one mitochondrial haplotype in 17 colonies from St. Croix, U.S. Virgin Islands. This high diversity suggests that *O. biroi* is native to Bangladesh, and our phylogenetic data indicate that the globally invasive lines originally stem from Bangladesh.

Our results confirm that *O. biroi* had already become invasive when it was originally described from Singapore in 1907 [15,23]. These samples were collected by Lajos Bíró following his travels to New Guinea from 1896 to 1902, so *O. biroi* must have become invasive before 1902 [24]. It is likely that *O. biroi* spread initially from Bangladesh via the historically important Bay of Bengal shipping ports, Dacca (present-day Dhaka) and Chittagong [25]. The Chittagong port existed at least as early as the second century, and both ports became major sources of international shipping activity from the 1600s beyond the 1800s [25]. Unfortunately, our present data do not provide a precise estimate for which port(s) and time periods might be responsible for the export of *O. biroi* from Bangladesh.

Invasive lines of *O. biroi* share a few traits with other invasive ant species that may allow them to thrive in human-modified habitats. These include parthenogenesis, retention of heterozygosity and the presence of multiple egg-layers within a colony [11,13]. Interestingly, we observed these three traits in the new *O. biroi* lines from the native range in Bangladesh (Lines I, J, K, L and M), which were not phenotypically distinct from the previously described lines from the invasive range (Lines A, B, C and D). Foucaud *et al.* [3] proposed a two-step model for the evolution of invasive ants: populations may adapt first to human-modified habitats within their native range and then spread to similar anthropogenic habitats around the world. Under this view, parthenogenesis, retention of heterozygosity and the presence of multiple egg-layers in *O. biroi* may all represent adaptations for survival in human-modified habitats in Bangladesh. Indeed, all three traits are found in invasive, but not native, populations of the little fire ant *Wasmannia auropunctata*. Evolution of parthenogenesis and polygyny in human-modified habitats possibly also occurred in *Mycocepurus smithii* and *Solenopsis geminata* [26–28].

In the light of the above case studies, we propose the following scenario. Prior to major anthropogenic impact, native populations in Bangladesh largely reproduced sexually, as do most ants, but had a propensity for asexual reproduction at some rate. As the native habitat became increasingly modified by humans, a larger subset of asexual genotypes were reproductively successful and became locally prolific in anthropogenic habitats. From that population, four genotypes were transported out of Bangladesh and ultimately became globally invasive. This model predicts that extant populations of *O. biroi* in undisturbed habitats may still reproduce sexually at some rate. We might also expect to find sexual reproduction in closely related *Ooceraea* species provided they have not experienced parallel evolution of parthenogenesis. The alternative scenario is that *O. biroi* was strictly asexual even before humans began transforming their habitat in Bangladesh. This seems unlikely, however, because automixis with central fusion leads to the gradual loss of heterozygosity. The fact that all genotyped samples of *O. biroi* are still highly heterozygous therefore suggests that they belong to relatively young asexual lineages, as is the case in several other invasive ants [3,13,26,27]. Genetic data from undisturbed habitats in Bangladesh and surrounding areas will be required to distinguish more definitively between these two possibilities.

## Supplementary Material

Table S1

## Supplementary Material

Table S2

## Supplementary Material

Supplementary Methods

## Data accessibility

All raw data for this article are available in electronic supplementary material, table S1 and table S2, and GenBank accession numbers provided therein.
