# Supplementary material for: Globally invasive populations of the clonal raider ant are derived from Bangladesh
Source: Biol Lett. 2020 Jun 17;16(6):20200105. doi: 10.1098/rsbl.2020.0105 (PMC7336853; doi:10.1098/rsbl.2020.0105)
Supplement: Table S1 [file rsbl20200105supp1.doc]

| ­Trible *et al.*  2020 samples | Clone Line | Locality | GPS Coordinates | Colony Size | *COI* | *COII* | *Wg* | *EF1α* | *LR* |
| --- | --- | --- | --- | --- | --- | --- | --- | --- | --- |
|  |  |  |  |  | 600 bp | 535 bp | 337 bp | 465 bp | 493 bp |
| BG1 | Line D | Khulna, Bangladesh | 89.53342, 22.80160 | 6 | MT086804 | MT086821 | MT086772 | MT086789 | MT086756 |
| BG2 | Line I | Khulna, Bangladesh | 89.50040, 22.90093 | 399 | MT086805 | MT086822 | MT086773 | MT086790 | MT086757 |
| BG3 | Line J | Khulna, Bangladesh | 89.48005, 22.95463 | 338 | MT086806 | MT086823 | MT086774 | MT086791 | MT086758 |
| BG5 | Line D | Jessore, Bangladesh | 89.20588, 23.16594 | 1 | MT086807 | MT086824 | MT086775 | MT086792 | MT086759 |
| BG6 | Line D | Jessore, Bangladesh | 89.20519, 23.16547 | 1 | MT086808 | MT086825 | MT086776 | MT086793 | MT086760 |
| BG7 | Line D | Jessore, Bangladesh | 89.20409, 23.16894 | 221 | MT086809 | - | MT086777 | MT086794 | MT086761 |
| BG8 | Line D | Jessore, Bangladesh | 89.20424, 23.16932 | 824 | MT086810 | MT086826 | MT086778 | MT086795 | MT086762 |
| BG9 | Line D | Jessore, Bangladesh | 89.20447, 23.16924 | 54 | MT086811 | MT086827 | MT086779 | MT086796 | MT086763 |
| BG10 | Line C | Jessore, Bangladesh | 89.06833, 23.08923 | 15 | MT086812 | MT086828 | MT086780 | MT086797 | MT086764 |
| Trible *et al.*  2020 samples | Clone Line | Locality | GPS Coordinates | Colony Size | *COI* | *COII* | *Wg* | *EF1α* | *LR* |
|  |  |  |  |  | 600 bp | 535 bp | 337 bp | 465 bp | 493 bp |
| BG11 | Line D | Jessore, Banlgadesh | 88.92284, 23.05006 | 783 | MT086813 | - | MT086781 | - | MT086765 |
| BG12 | Line K | Dhaka, Bangladesh | 90.39161, 23.73577 | 77 | MT086814 | - | MT086782 | MT086798 | MT086766 |
| BG13 | Line L | Lawachara, Bangladesh | 91.76547, 24.29717 | 67 | MT086815 | MT086829 | MT086783 | MT086799 | MT086767 |
| BG14 | Line M | Lawachara, Bangladesh | 91.76439, 24.30053 | 370 | MT086816 | MT086830 | MT086784 | MT086800 | - |
| BG15 | Line M | Lawachara, Bangladesh | 91.76439, 24.30053 | 9 | MT086817 | - | MT086785 | - | MT086768 |
| BG16 | Line M | Lawachara, Bangladesh | 91.76657, 24.30108 | 54 | MT086818 | MT086831 | MT086786 | MT086801 | MT086769 |
| Shen1 | Line C | Shenzen, China | - | - | MT086819 | MT086832 | MT086787 | MT086802 | MT086770 |
| Viet1 | Line D | Ba Vì, Vietnam | - | - | MT086820 | - | MT086788 | MT086803 | MT086771 |
| Sing1 | Line B | Singapore | - | - | MT108294 | MT108295 | MT108298 | MT108296 | MT108297 |
|  |  |  |  |  |  |  |  |  |  |
|  |  |  |  |  |  |  |  |  |  |
|  |  |  |  |  |  |  |  |  |  |
| Kronauer *et al.* 2012 samples | Colony code | Locality | GPS Coordinates | Colony Size | *COI* | *COII* | *Wg* | *EF1α* | *LR* |
|  |  |  |  |  | 600 bp | 535 bp | 337 bp | 465 bp | 493 bp |
| C13 | Line A | Okinawa, Japan | - | - | JX157194 | JX157205 | JX157222 | JX157214 | JX157228 |
| STC1 | Line B | Jolly Hill,  St. Croix | - | - | JX157200 | JX157211 | JX157226 | JX157220 | JX157234 |
| C11 | Line C | Okinawa, Japan | - | - | JX157193 | JX157204 | JX157221 | JX157213 | JX157227 |
| Cbi48 | Line D | Tutuila, American Samoa | - | - | JX157201 | JX157212 | - | - | - |
| Cbi25 | Genotype E | Uttarakhand, India | - | - | JX157196 | JX157207 | JX157224 | JX157216 | JX157230 |
| Cbi26 | Genotype F | Jammu, India | - | - | JX157197 | JX157208 | - | JX157217 | JX157231 |
| Cbi6 | Genotype G | Nghệ An, Vietnam | - | - | JX157195 | JX157206 | JX157223 | JX157215 | JX157229 |
| Kronauer *et al.* 2012 samples | Colony code | Locality | GPS Coordinates | Colony Size | *COI* | *COII* | *Wg* | *EF1α* | *LR* |
|  |  |  |  |  | 600 bp | 535 bp | 337 bp | 465 bp | 493 bp |
| Cbi27 | Genotype H | Guangdong, China | - | - | JX157198 | JX157209 | - | JX157218 | JX157232 |
| Outgroup |  |  |  |  |  |  |  |  |  |
| *O. australis* | PSW#15702 | Cape York, Australia | - | - | JX157199 | JX157210 | JX157225 | JX157219 | JX157233 |

|  |  |
| --- | --- |
