# Supplementary material for: Globally invasive populations of the clonal raider ant are derived from Bangladesh
Source: Biol Lett. 2020 Jun 17;16(6):20200105. doi: 10.1098/rsbl.2020.0105 (PMC7336853; doi:10.1098/rsbl.2020.0105)
Supplement: Supplementary Methods [file rsbl20200105supp3.docx]

**Supplementary Methods:** Globally invasive populations of the clonal raider ant are derived from Bangladesh

Waring Trible*^1,2^, Sean K. McKenzie^1,3^, Daniel J. C. Kronauer*^1^

Field colonies of *O. biroi* from Bangladesh were collected by hand in October and November of 2014, and maintained in the laboratory to test whether they could propagate in the absence of sexual reproduction. Colonies collected in Bangladesh were counted in the field to determine colony sizes, and dead ants were preserved in 95% ethanol for DNA extraction and genotyping. Additional samples for genotyping were obtained from Singapore, China, and Vietnam.

Genomic DNA was extracted using Qiagen’s QIAmp DNA Micro Kit following manufacturer’s recommendations. For phylogenetic analysis, primers for DNA sequencing were as in Kronauer *et al.* 2012, and Sanger sequencing was performed by Macrogen (New York, NY). DNA fragments were PCR amplified in a final volume of 12 μl using Applied Biosystems’ AmpliTaq Gold Kit following manufacturer’s recommendations. Cycling profiles started with a denaturation at 94 °C for 10 min, and then proceeded with 40 cycles of 94 °C for 30 s, 55 °C annealing for 30 s, and 72 °C extension for 30 s, followed by a final extension step of 72 °C for 10 min.

Phylogenetic analyses were performed using new and published (Kronauer *et al.* 2012) sequences. Nucleotide sequences were aligned using MUSCLE in MEGA 7, and tree topology was estimated using Maximum Likelihood analysis in RAxML version 8 with the GTR + GAMMA substitution model.

Under automixis with central fusion and low levels of recombination, we expected most nestmates to possess identical multi-lo­cus genotypes. Furthermore, most or all of the genotypic variation that does exist should be consistent with losses-of-heterozygosity due to rare recombination events during meiosis I (Kronauer *et al.* 2012). To test for clonality, 5-7 individuals were genotyped from nine exemplar Bangladesh colonies. Bangladesh 14, which was collected as a single individual, was excluded. Consistency with clonal reproduction was therefore established if most individuals in each colony possessed identical genotypes, and all within-colony genetic variation could be explained by loss-of-heterozygosity events. We were not able to employ the clonality statistics used in Kronauer *et al.* 2012, as these require testing statistically independent populations. Here, the majority of our data come from non-independent populations in Bangladesh (Stenberg *et al.* 2003; Arnaud-Haond *et al.* 2007).

Five conserved microsatellite loci were used for population genetic analysis: Ant20, Ant859, Ant1343, Ant2794, and Ant4155 (Butler *et al.* 2014). Reference specimens from Lines A, B, C, and D from Kronauer *et al.* 2012 were also genotyped for these loci. Fragment analysis was performed by GeneWiz (Frederick, MD). Allele calling was performed using Peak Scanner (Applied Biosystems). Failed or ambiguous reactions were repeated up to three times to ensure accuracy and consistency. Reagents and cycling profiles followed Butler *et al.* 2014.

**References**

Arnaud-Haond S, Duarte CM, Alberto F, Serrão EA. 2007 Standardizing methods to address clonality in population studies. *Mol. Ecol.* **16**, 5115–5139. (doi:10.1111/j.1365-294X.2007.03535.x)

Butler IA, Siletti K, Oxley PR, Kronauer DJC. 2014 Conserved microsatellites in ants enable population genetic and colony pedigree studies across a wide range of species. *PLoS One* **9**, e107334. (doi:10.1371/journal.pone.0107334)

Kronauer DJC, Pierce NE, Keller L. 2012 Asexual reproduction in introduced and native populations of the ant *Cerapachys biroi*. *Mol. Ecol.* **21**, 5221–5235. (doi:10.1111/mec.12041)

Stenberg P, Lundmark M, Saura A. 2003 MLGsim: A program for detecting clones using a simulation approach. *Mol. Ecol. Notes* **3**, 329–331. (doi:10.1046/j.1471-8286.2003.00408.x)
